# Histological Characterisation of a Sheep Model of Mild Traumatic Brain Injury: A Pilot Study

**DOI:** 10.1089/neur.2023.0105

**Published:** 2024-03-06

**Authors:** Sheryl Tan, Danica Hamlin, Eryn Kwon, Miriam Scadeng, Vickie Shim, Samantha Holdsworth, Sarah-Jane Guild, Helen Murray

**Affiliations:** ^1^ Centre for Brain Research, University of Auckland, Grafton, New Zealand.; ^2^ Department of Anatomy and Medical Imaging, University of Auckland, Grafton, New Zealand.; ^3^ Mātai Medical Research Institute, Gisborne, New Zealand.; ^4^ Auckland Bioengineering Institute, Grafton, New Zealand.

**Keywords:** hemorrhage, immunohistochemistry, mild traumatic brain injury, sheep, neuroinflammation

## Abstract

Large animal models of mild traumatic brain injury (mTBI) are needed to elucidate the pathophysiology of mechanical insult to a gyrencephalic brain. Sheep (ovis aries) are an attractive model for mTBI because of their neuroanatomical similarity to humans; however, few histological studies of sheep mTBI models have been conducted. We previously developed a sheep mTBI model to pilot methods for investigating the mechanical properties of brain tissue after injury. Here, we sought to histologically characterize the cortex under the impact site in this model. Three animals received a closed skull mTBI with unconstrained head motion, delivered with an impact stunner, and 3 sham animals were anesthetized but did not receive an impact. Magnetic resonance imaging (MRI) of the brain was performed before and after the impact and revealed variable degrees of damage to the skull and brain. Fluorescent immunohistochemistry revealed regions of hemorrhage in the cortex underlying the impact site in 2 of 3 mTBI sheep, the amount of which correlated with the degree of damage observed on the post-impact MRI scans. Labeling for microtubule-associated protein 2 and neuronal nuclear protein revealed changes in cellular anatomy, but, unexpectedly, glial fibrillary acidic protein and ionized calcium-binding adaptor molecule 1 labeling were relatively unchanged compared to sham animals. Our findings provide preliminary evidence of vascular and neuronal damage with limited glial reactivity and highlight the need for further in-depth histological assessment of large animal mTBI models.

## Introduction

Animal models of traumatic brain injury (TBI) are essential for studying the underlying pathophysiology of mechanical insult to the brain. Although lissencephalic species such as rodents are commonly used to study disease processes and affected pathways, they cannot reproduce the shear strain experienced by a gyrencephalic brain.^1–3^ Complementary studies in large animals can facilitate more representative models that account for the size and gyrencephalic structure of the human brain. Domestic sheep (ovis aries) are becoming a popular animal model in biomedical research because of their docile nature, long life span, and neuroanatomical similarity to humans in terms of white matter volume, brain size, and gyrencephalic structure.^4^ Understanding the effect of injury on the gyrencephalic brain is becoming increasingly important because of the growing awareness of chronic traumatic encephalopathy and the specific sulcal tau lesions that form in persons with a history of repeated TBI.^1^

We previously conducted a pilot study of closed-head mild TBI (mTBI) with unconstrained head motion in sheep to facilitate biomechanical testing of brain tissue. Compression mechanical testing was conducted to assess baseline regional variations in material properties and how these properties changed after mechanical impact. We found that compressive strength was reduced in the area nearest the direct impact.^5^ We used magnetic resonance imaging (MRI) data from these sheep to validate a finite element model that accurately predicted the damage pattern observed on post-impact MRI scans.^6,7^

Here, we sought to histologically characterize the region under the impact site from the sheep in this pilot study to examine whether changes in cellular anatomy or inflammatory status were induced in this model. Previous studies of sheep TBI models demonstrate that diffuse axonal injury is observed 4 h post-impact,^8,9^ and immunoreactivity for microtubule-associated protein 2 (MAP2), a marker of the neuronal cytoskeleton, is reduced 2 h post-impact.^10^ Therefore, we hypothesized that changes in cellular anatomy and inflammation could be present at the acute 3-h post-impact time point of our study, thus providing insights into the early pathological processes occurring in a large animal mTBI model.

## Methods

### Animals

A total of 6 sheep were available for this study: 3 sham animals and 3 acutely impacted animals. All sheep were ewes of the mixed Romney breed, between 3 and 5 years old. Ethical approval for the study was granted by the University of Auckland Animal Ethics committee (AEC approval no.: 002170), and protocols were carried out in accordance with the New Zealand Animal Welfare Act 1999. All animal-related procedures were performed under anesthesia by trained and licensed staff. Animals were anesthetized with isoflurane and remained under anesthesia for a total of 5 h. They received a baseline MRI scan of ∼2 h. More than 18 different scanning sequences were used to acquire baseline functional, physiological, and structural information of the brain. These sequences include T1 magnetization-prepared rapid gradient-echo for gathering structural information and T2 fluid-attenuated inversion recovery for white matter abnormalities and injury information, and high angular resolution diffusion imaging for capturing white matter fiber tracts. These data were analyzed in our previous publications.^5,6^

After the baseline scan, the animal remained under anesthesia for the impact delivery. The acute impact was delivered to the animal using a CASH^®^ Special Concussion stunner, with a one-grain cartridge that produces ∼76 J of impact energy (as provided by the manufacturer, section 5 of the User Manual) from a flat, circular impactor (ø = 25 mm). The impact delivery was dynamic, resulting in a non-penetrating injury from a direct impact with unconstrained head motion comparable to previous ovine models of TBI.^11,12^ The animal was in a sphinx position, with its head supported by a pillow to allow natural recoil movement. The stunner was positioned perpendicular to the area between the horn buds, centered above the midsagittal plane. On the brain, this resulted in an impact on the superior frontal area of the cerebrum. After the impact, the animal underwent a second MRI scan identical to the baseline sequence.

### Tissue processing

At the end of the imaging session, ∼3 h after impact, animals were euthanized with pentobarbital, and the brain was removed and chilled at 4°C for 30 min.^13^ The brain was then placed in a holder specifically designed to fit the lower half of the brain with a 5-mm cutting guide in the coronal plane.^5^ The entire brain was sectioned in 5-mm-thick slices using a microtome blade and separated into left and right hemispheres. Cut slices were submerged in 1 × phosphate-buffered saline (PBS) at room temperature. For the mechanical testing previously reported,^5^ a 20 × 20 mm square sample was cut from each slice for both hemispheres adjacent to the medial line and as close as possible to the impact site. This left a small sample of gyrus on the dorsal surface of each slice for histological analysis.

Tissue from directly under the region of impact was selected for this study. Tissue blocks were immersion-fixed with 15% formalin for 96 h at room temperature. Next, specimens were dehydrated in a graded alcohol series and cleared in chloroform in an automated tissue processor (Leica APS300S; biopsy cycle; Leica Biosystems, Danvers, MA), followed by embedding in paraffin wax (Leica Paraplast, 39601006; Leica Biosystems). Paraffin-embedded tissue blocks were cut at a thickness of 10 μm on a rotary microtome (Leica Biosystems RM2235; Leica Biosystems). Sections were floated in a heated (40°C) water bath and mounted on SuperFrost Plus^TM^ (Menzel-Gläser).

### Multiplexed immunohistochemistry

Because of the limited amount of tissue from the lesion area available for immunohistochemistry, we conducted multiplexed immunohistochemistry to screen for differences in a range of markers, including ionized calcium-binding adaptor molecule 1 (Iba1), glial fibrillary acidic protein (GFAP), neuronal nuclear protein (NeuN), MAP2, myeloperoxidase (MPO), collagen IV, and amyloid precursor protein (APP). This technique allows for multiple markers to be labeled on the same tissue section, thereby maximizing the amount of information acquired from the available tissue.

One section from each animal was processed for multiplexed fluorescence immunohistochemistry, using iterative cycles of antibody labeling as previously described.^14,15^ Sections were deparaffinized and subjected to heat-mediated epitope retrieval using 10 mM of Tris/EDTA buffer (pH 9.0) in a pressure cooker (2100 Antigen Retriever; Aptum Biologics Ltd, Southampton, UK). TrueBlack reagent (Biotium, Inc., Fremont, CA) was applied as per the manufacturer's instructions to quench endogenous autofluorescence, and 10% normal goat serum diluted in PBS was subsequently applied for 1 h at room temperature to block non-specific binding of the secondary antibody. Sections were then incubated overnight in the round 1 primary antibody cocktail (chicken polyclonal Iba1; Synaptic Systems 195-005; 1:1000; Synaptic Systems GmbH, Goettingen, Germany), guinea pig polyclonal NeuN (Merck ABN90P; 1:1000; MilliporeSigma, Burlington, MA), diluted in 1% normal goat serum in PBS at 4°C. Sections were subsequently incubated in the appropriate species-specific secondary antibody cocktail (goat antichicken AF488, 1:500 [Abcam ab150173; Abcam, Cambridge, MA]; goat anti-guinea-pig AF546, 1:500 [ThermoFisher A11074; ThermoFisherScientific, Waltham, MA], and containing Hoechst 33342 (ThermoFisher H1399, 1:10,000; ThermoFisherScientific) to stain nuclei, for 3 h at room temperature. Sections were washed in PBS between all steps.

All slides were cover-slipped with Slowfade Diamond antifade mountant (ThermoFisher) and imaged on an automated wide-field fluorescence microscope (Zeiss Z2 Axioimager; Carl Zeiss, Oberkochen, Germany) equipped with MetaSystems Metafer and Vslide software, with a 10 × air objective (0.9 numerical aperture). Sections were labeled in the same batch to maintain consistency, and the image acquisition settings were kept the same for each marker across all sections. One channel with no labeling was acquired in each round to capture the autofluorescence in the section. After imaging, the cover-slip was removed by immersion in PBS, and the antibodies were stripped from the tissue by applying 5X NewBlot™ Nitro Stripping Buffer (undiluted; LI-COR Biosciences, Lincoln, NE) for 10 min at room temperature. The effectiveness of this antibody stripping protocol is demonstrated in Supplementary Figure S1. The section was rewashed in PBS, and subsequent rounds of labeling and imaging were performed as above. Round 2 antibodies were as follows: rabbit polyclonal MPO (Agilent A0398; 1:1000; Agilent Technologies, Lexington, MA) and chicken polyclonal MAP2 (Abcam ab5392; 1:1000); corresponding secondary antibodies were goat antirabbit AF546 (ThermoFisher A11035; 1:500; ThermoFisherScientific) and goat antichicken AF647 (ThermoFisher A21449; 1:500; ThermoFisherScientific).

Round 3 antibodies were as follows: rabbit polyclonal collagen IV (Abcam ab6586; 1:500) and chicken polyclonal GFAP (Abcam 4674; 1:1000); corresponding secondary antibodies were: goat antirabbit AF647 (ThermoFisher, A21245; 1:500; ThermoFisherScientific) and goat antichicken AF546 (ThermoFisher A11040; 1:500; ThermoFisherScientific). In total, three rounds of labeling were performed. Images from all rounds were aligned using a custom Python script.^16^

### Single-round immunofluorescent triple labeling

We processed sequential sections from each animal for standard fluorescent labeling with each antibody to validate the antibody labeling observed with the multiplex immunohistochemistry protocol. Labeling and imaging were carried out using the same protocol as for the multiplex immunohistochemistry, with the omission of subsequent rounds of labeling. Note that APP labeling (Merck MAB384; 1:100; MilliporeSigma) was conducted using standard fluorescent labeling only.

### Image analysis

The single-round and multiplex labeled sections from each animal were quantitatively analyzed. All measurements and cell counts were made using ImageJ (v1.54f; National Institutes of Health, Bethesda, MD) on 8-bit grayscale TIFF images. A region of gray matter (∼3 mm^2^) encompassing all six cortical layers was first delineated using the Hoechst image. Regions of hemorrhage were then masked using the autofluorescence image. First, a binary mask was created using the threshold tool with a lower threshold value of 30. The binary “fill holes” function was then applied, followed by the Analyze Particles function (minimum size of 2000 pixels). This mask was then subtracted from each of the antibody images to exclude the regions of hemorrhage from the analysis.

For each antibody image, a 100-pixel rolling ball background subtraction was then applied to remove the non-specific background signal. A binary mask was then created using the threshold tool and a lower threshold value of 10. The number of pixels within the thresholded area was measured and divided by the total number of pixels in the image to give the percentage area of the image labeled by the antibody.

MPO-positive cells were manually counted using the multi-point selection tool in ImageJ because the number of objects was relatively small. The MPO image was overlaid with the collagen IV image, and cells were manually classified as extravascular if they did not colabel with a collagen IV–positive blood vessel.

We did not detect any qualitative differences between the single-round labeling and the multiplexed labeling for any marker. Similarly, there was <10% difference in values for the quantitative measures between the single-round labeling and multiplexed labeling for all markers. Therefore, we have reported the mean value of the two sections for all quantitative measures.

## Results

### Hemorrhage and extravasation of neutrophils after mild traumatic brain injury

**FIG. 1.**
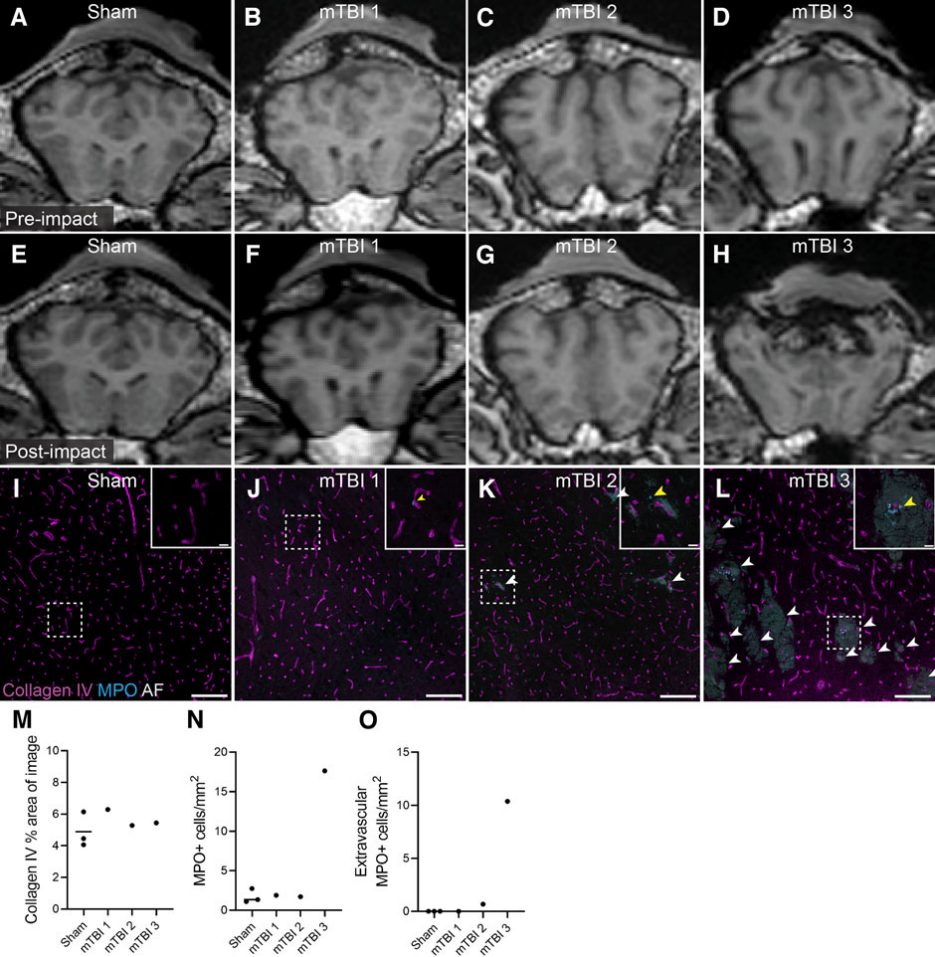
Macroscopic damage and vessel pathology in sheep model of mTBI. Representative coronal T1-weighted MR images of the brain before impact for a sham (**A**) and the 3 mTBI sheep (**B–D**) and the corresponding post-impact images (**E–H**). Whereas mTBI sheep 1 or 2 showed no obvious skull fracture or hemorrhage (**F,G**), mTBI sheep 3 had significant skull fracture and brain hemorrhaging (**H**). (**I–L**) Representative images of immunohistochemical labeling for collagen IV and MPO, together with the autofluorescence (AF) channel, in the cortex underlying the impact site for a sham (**I**) and the 3 mTBI sheep (**J–L**). Scale bar = 200 μm. White arrows indicate areas of hemorrhage. Inset images depict higher magnification images of the regions indicated by the white box. Scale bar = 20 μm. MPO^+^ neutrophils (inset images, yellow arrows) are observed outside blood vessels in areas of hemorrhage (**K,L**). Quantification of the (**M**) mean percentage area of the image that is positive for collagen IV labeling, (**N**) mean number of MPO^+^ cells/mm^2^, and (**o**) extravascular MPO^+^ cells/mm^2^. Data are presented as individual points per animal. MPO, myeloperoxidase; MR, magnetic resonance; mTBI, mild traumatic brain injury.

The 3 mild TBI (mTBI) sheep were impacted with the same amount of energy (∼76 J) at a similar anatomical location, but showed varying degrees of brain trauma (Fig. 1A–H). The first sheep (mTBI sheep 1) suffered the most minor damage with only a superficial skin tear at the impact site and no skull fracture, edema, or hemorrhage observed in the post-impact MRI (Fig. 1B,F). The second sheep (mTBI sheep 2) showed regions of edema at the impact location, but no skull fracture or hemorrhage in the post-impact MRI (Fig. 1C,G). The third sheep (mTBI sheep 3) suffered the most damage, with evidence of skull fracture at the impact site and hemorrhage in the post-impact MRI (Fig. 1D,H). The cortical gray matter directly under the impact site showed areas of hemorrhage in mTBI sheep 2 and 3, observed as autofluorescent red blood cells accumulating around collagen IV–positive vessels. No hemorrhage was observed in the corresponding cortical regions of the 3 sham animals (Fig. 1i).

In the 3 mTBI sheep, microscopically, the degree of hemorrhage observed corresponded with the relative degree of macroscopic brain trauma. No areas of hemorrhage were observed histologically in mTBI sheep 1 (Fig. 1J). For mTBI sheep 2, small, discrete regions of hemorrhage were observed in the cortical gray matter underlying the impact site (Fig. 1K). For mTBI sheep 3, large regions of hemorrhage were observed in the cortical gray matter underlying the impact site (Fig. 1L). The area of collagen IV–positive blood vessels was similar between the 3 sham and 3 mTBI animals, suggesting no overt difference in vessel coverage (Fig. 1M). The number of MPO-positive neutrophils observed in the cortical gray matter underlying the impact site was the highest in mTBI sheep 3 (Fig. 1N), and these neutrophils were predominantly observed outside collagen IV–positive vessels within areas of hemorrhage, indicating extravasation (Fig. 1L,O).

### NeuN- and MAP2-positive cells have altered morphology after mTBI, but GFAP and Iba1 labeling is unchanged

**FIG. 2.**
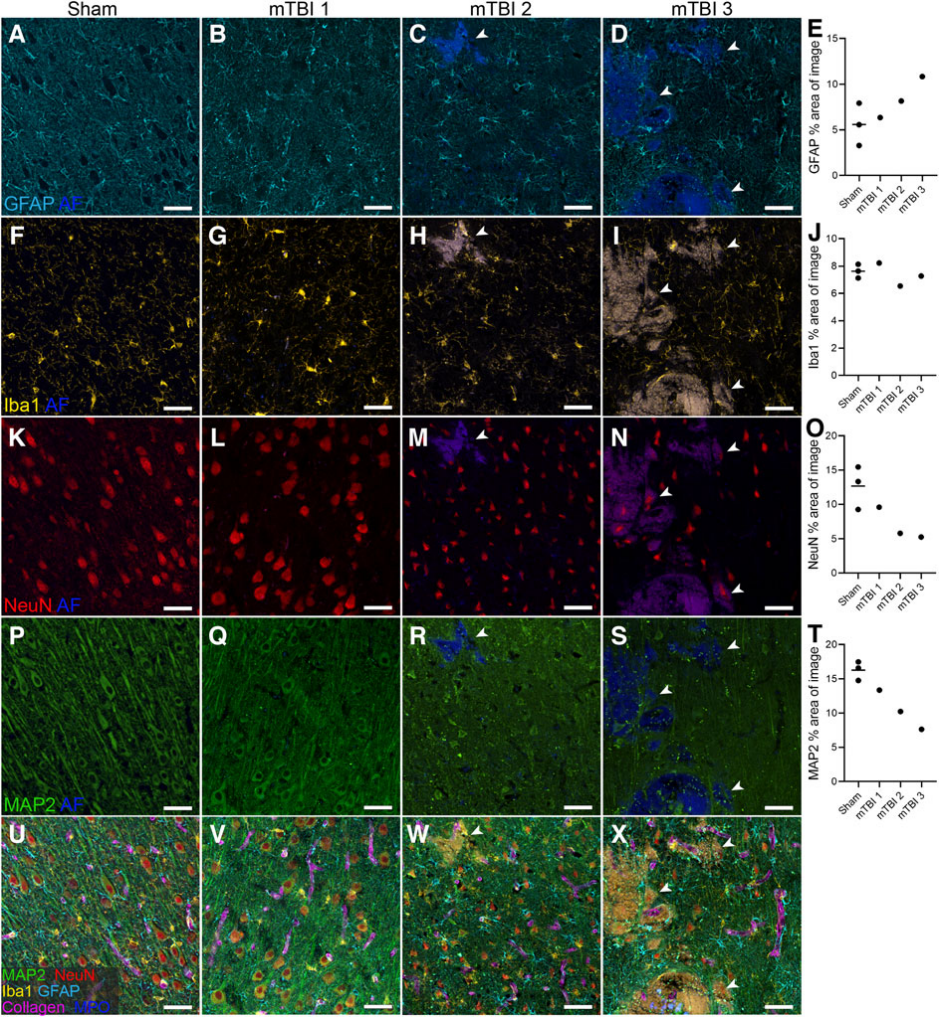
Markers of astrocytes, microglia, and neurons in a sheep model of mTBI. Representative images of immunohistochemical labeling for GFAP (**A–D**), Iba1 (**F–I**), NeuN (**K–N**), and MAP2 (**P–S**) together with the autofluorescence (AF) channel in the cortex underlying the impact site for 1 sham and the 3 mTBI sheep. Images of the multiplexed labeling from all markers for each sheep (**U–X**). Scale bar = 50 μm. White arrows indicate areas of hemorrhage. Quantification of the mean percentage area of the image that is positive for GFAP (**E**), Iba1 (**J**), NeuN (**O**), and MAP2 (**T**) is presented as individual points per animal. GFAP, glial fibrillary acidic protein; Iba1, ionized calcium-binding adaptor molecule 1; MAP2, microtubule-associated protein 2; MPO, myeloperoxidase; mTBI, mild traumatic brain injury; NeuN, neuronal nuclear protein.

To examine the neuroinflammatory status of the area underlying the impact site, we examined labeling for the astrocyte marker GFAP and the microglial marker Iba1. There was no overt difference in the morphology of GFAP-positive cells in the 3 mTBI animals compared to the sham animals (Fig. 2A–D). GFAP-positive cells clustered around the areas of hemorrhage in mTBI sheep 3, the animal that sustained the most damage, but this was not apparent in mTBI sheep 2, which had less hemorrhage. The percentage area of GFAP labeling was highest in mTBI sheep 3, followed by mTBI sheep 2 and mTBI sheep 1 (Fig. 2E). Similarly, there was no overt difference in the morphology of Iba1-positive cells in the 3 mTBI animals compared to the sham animals (Fig. 2F–I); however, these cells did appear to cluster around the areas of hemorrhage in both mTBI sheep 2 and 3. Quantitative assessment of the percentage area of Iba1 labeling showed similar coverage of Iba1 between sham and mTBI sheep (Fig. 2J).

To examine the effect on neurons in the area underlying the impact site, we examined labeling for the neuronal markers NeuN and MAP2. No qualitative difference in NeuN-positive cell morphology was observed in mTBI sheep 1, the animal with no microscopic hemorrhage, compared to the shams (Fig. 2K–L). However, the NeuN-positive cells in mTBI sheep 2 and 3 were smaller than those of the sham animals (Fig. 2M,N and Supplementary Fig. S2). The percentage area of NeuN labeling was lowest for mTBI sheep 2 and 3 compared to mTBI sheep 1 and the shams (Fig. 2O). MAP2 immunoreactivity was similar in mTBI sheep 1 compared to the shams; however, in mTBI sheep 2 and 3, the labeling was sparse and noticeably fewer cytoplasmic and axonal structures were observed (Fig. 2P–S). Quantitatively, the percentage area of MAP2 labeling was lower in the mTBI sheep compared to the sham group (Fig. 2T). We did not observe any labeling for APP, a marker of impaired axonal transport in either the sham or mTBI sheep (Supplementary Fig. S3). When the labeling from all multiplexed markers was overlaid on the same image, we did not detect clustering of Iba1^+^ microglia or GFAP^+^ astrocytes around areas of hemorrhage (Fig. 2U–X).

## Discussion

Despite growing recognition of the value of large animal models for TBI research, limited histological studies of these models have been published. Therefore, in this study, we sought to perform a histological characterization of the cortex under the impact site in tissue from mTBI sheep that were previously used to pilot methods for investigating the mechanical properties of brain tissue after injury.^5–7^ We identified areas of hemorrhage in 2 of the 3 mTBI sheep, the amount of which correlated with the degree of damage observed on the post-impact MRI scans. Labeling for MAP2 and NeuN revealed changes in cellular anatomy for the 2 sheep that had regions of hemorrhage, but, perhaps unexpectedly, GFAP and Iba1 labeling were relatively unchanged in these animals compared to the sham animals and the mTBI sheep without hemorrhage. Our findings provide histological evidence of blood vessel and neuronal damage with limited glial reactivity concordant with reports from previous large animal mTBI models.^8,10,17^ While this pilot study used a small number of animals, our findings highlight the need for further in-depth histological assessment of large animal mTBI models and demonstrate the potential of this model for future studies of mTBI pathophysiology.

Reduced MAP2 labeling and the appearance of shrunken NeuN-positive cells suggest structural neuronal damage and altered neuronal function.^18^ Of note, we only observed these alterations in cellular anatomy in the animals where hemorrhage was detected, which may suggest that a minimum threshold of damage is necessary before neuronal cytoskeletal changes can be observed. Given that we collected the brain tissue within 3 h of the impact, these morphological changes may represent the early stages of cell death, or these cells may eventually recover. Further studies examining varying survival times post-injury would be needed to elucidate this.

Reduced MAP2 labeling in the cortex underlying the impact site was also observed in a previous mTBI sheep model of direct impact injury^10^ and aligns with previous reports of axonal injury in mTBI sheep models,^8,9,12^ pig models,^17,19,20^ and in a rat TBI model.^18^ However, we did not observe APP^+^ axons, a marker of impaired axonal transport, in the cortex underlying the impact site in our model. This is somewhat in agreement with previous studies that examined APP immunoreactivity in similar cortical regions at this acute time point. Whereas rotational injury pig models demonstrate the presence of APP^+^ axons,^19^ a recent semiquantitative analysis of APP labeling in this model found no significant change compared to sham animals at 1 day post-injury.^17^ A previous study of APP labeling in a sheep model of direct impact injury at a 4-h time point found minimal APP^+^ axons and no significant difference between injured and sham animals. However, an increase in axons labeled by the 1176-residue calpain-derived alpha-II spectrin N-terminal fragment (SNTF) was detected.^8^ This marker is proposed to reflect injury-induced elevations in axonal calcium and activation of calcium-dependent proteases (calpains).^21^ SNTF^+^ axons were observed at 6, 48, and 72 h post-injury in a pig TBI model.^21^

Although the mechanism of MAP2 loss is unknown, previous studies have suggested that an excitotoxic loss of calcium homeostasis and subsequent calpain activation may lead to cytoskeletal degeneration.^10,18^ In summary, though we observed axonal damage based on MAP2 labeling in the cortex underlying the impact site 3 h post-injury in our model, this was not accompanied by an increase in APP labeling. It should be noted that we did not examine other white matter regions in our study, and other markers such as SNTF may be needed to provide a more comprehensive assessment of axonal injury in large animal mTBI models.

Our GFAP and Iba1 labeling showed no distinct differences in astrocyte and microglia morphology between the sham and mTBI sheep, even in those animals with substantial hemorrhage and neuronal damage. These findings echo those reported in a similar sheep impact model of mTBI with or without hypoxia.^8^ Conversely, in a pig rotational TBI model, rapid microglial reactivity was shown to occur specifically in proximity to injured neurons,^22^ and in mini-pig rotational TBI models microglial reactivity was detected in areas that also showed diffuse axonal injury.^17,20^ It is possible that the “percentage area of labeling” measure we used is not sensitive enough to detect subtle changes in glial morphology, given that changes in microglial structure were only detected in the mini-pig model when the ramification of microglial processes was assessed.^17,20^

Our sheep model also differed from these pig models in that it used a direct impact injury rather than a rotational injury. The acute time (∼3 h) between impact and brain collection in our study may also be too short to observe overt glial changes in a direct impact injury model. Whereas previous sheep TBI models have demonstrated that pathological changes such as diffuse axonal injury^8,9^ and reduced MAP2 immunoreactivity^10^ occur at acute time-points (2–4 h post-injury), our results suggest that overt glial recruitment does not occur in this time frame in a direct impact mTBI sheep model. Importantly, our GFAP and Iba1 labeling did not capture the functional changes that may have been occurring in these cells. Sheep-specific antibodies that can label proteins more indicative of microglial functions, such as phagocytosis or antigen presentation, are needed to provide a more nuanced assessment of inflammatory status. The lack of readily available antibodies for immunohistochemistry of sheep tissue is a limitation of this model.

The procedures for this study were primarily designed for piloting biomechanical testing of the tissue, which introduced limitations such as the limited amount of tissue available for immunohistochemistry and the acute 3-h time point. Further, the very low number of animals in the mTBI group and the variable degree of damage caused by the impact for each animal meant that we could not perform statistical analysis of our quantitative histological measures. Although this variability in damage could be observed as analogous to the diversity of injuries noted in human mTBI, it reinforces the need for a larger sample size for future histological studies of large animal models. Future studies should also aim to reconcile the timeline of histological changes observed in rodent and large animal mTBI models.

## Conclusion

In conclusion, from our histological analysis of the cortical gray matter underlying the impact site in a sheep mTBI model, we qualitatively observed changes in neuronal structure that correlated with the degree of macroscopic damage observed by gross inspection and MRI. However, no obvious astrocyte or microglial reactivity was observed, even in the animal with the most severe damage and largest area of hemorrhage. Our findings highlight the need for more species-specific antibodies to characterize the pathological processes occurring in large animal mTBI models so that we may bridge the insights from rodent models to human injury. This preliminary study demonstrates the potential of sheep as a model for studying mTBI pathophysiology.

## Supplementary Material

Supplemental data

Supplemental data

Supplemental data

## Data Availability

The data that support the findings of this study are available on request from the corresponding author.

## Notes

[Supplementary-material SD1]

[Supplementary-material SD2]

[Supplementary-material SD3]

## Abbreviations Used

APP: amyloid precursor protein
Iba1: ionized calcium-binding adaptor molecule 1
GFAP: glial fibrillary acidic protein
MAP2: microtubule-associated protein 2
mTBI: mild traumatic brain injury
MPO: myeloperoxidase
MRI: magnetic resonance imaging
NeuN: neuronal nuclear protein
PBS: phosphate-buffered saline
SNTF: spectrin N-terminal fragment
TBI: traumatic brain injury
