# Supplementary figures and images for: Histological Characterisation of a Sheep Model of Mild Traumatic Brain Injury: A Pilot Study
Source: Neurotrauma Rep. 2024 Mar 6;5(1):194–202. doi: 10.1089/neur.2023.0105 (PMC10924061; doi:10.1089/neur.2023.0105)

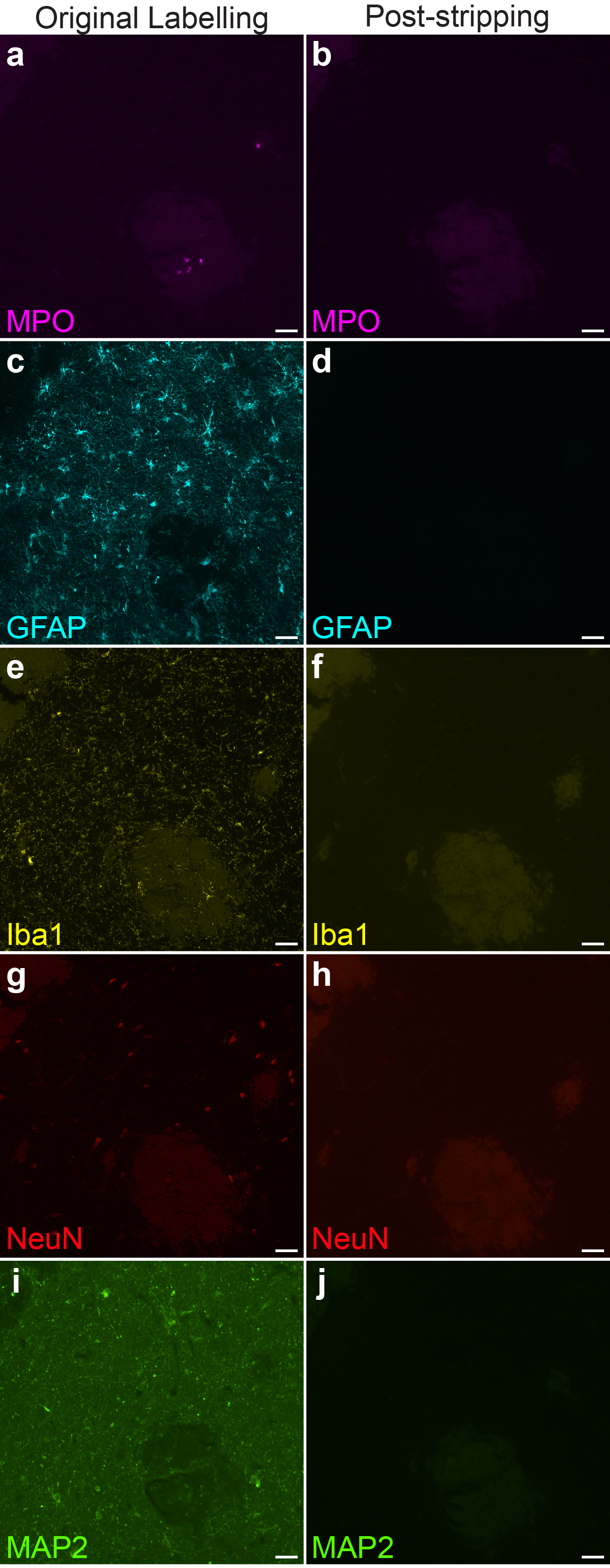

Supplement: Supplemental data [file Suppl_FigureS1.tif]

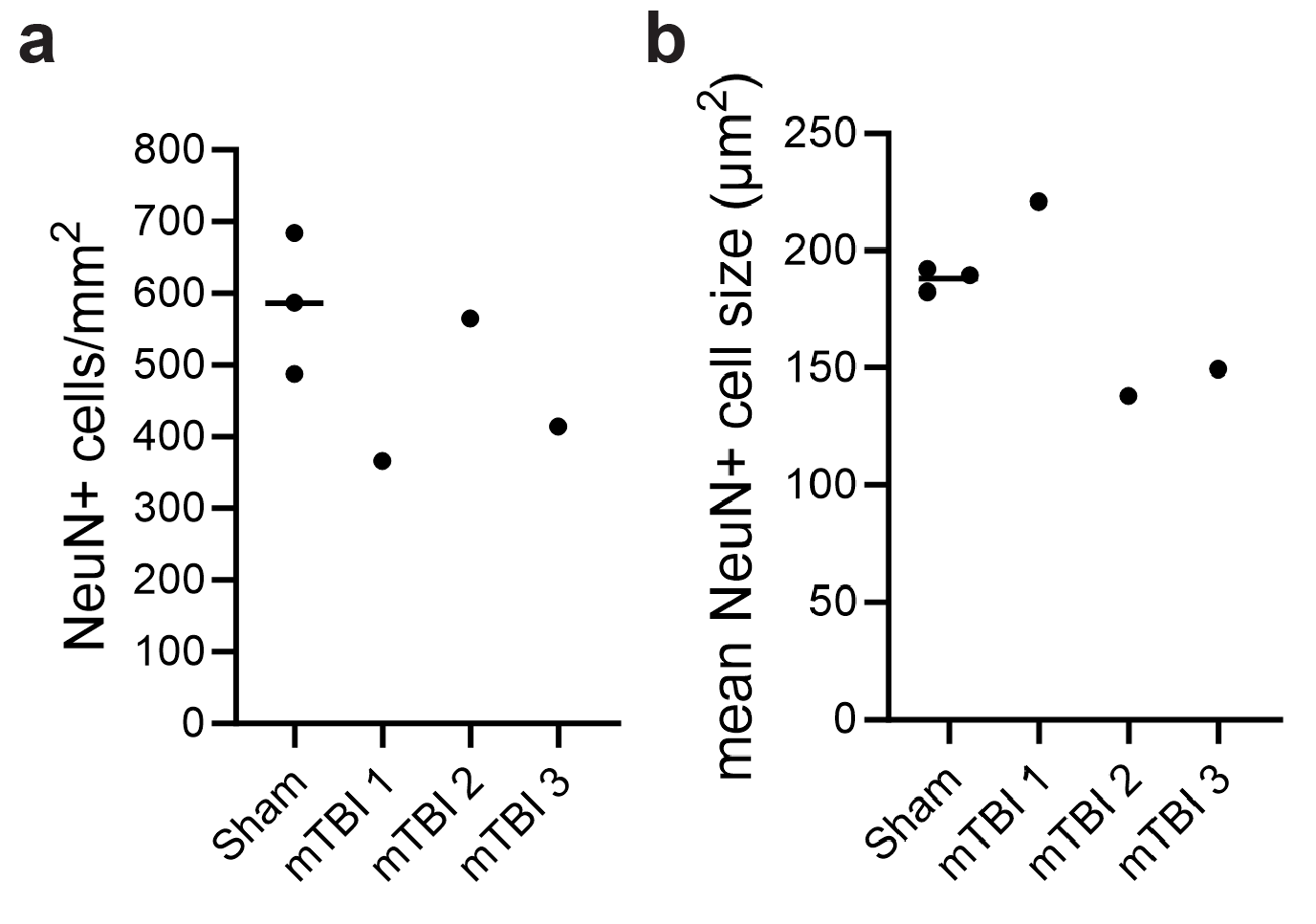

Supplement: Supplemental data [file Suppl_FigureS2.tif]

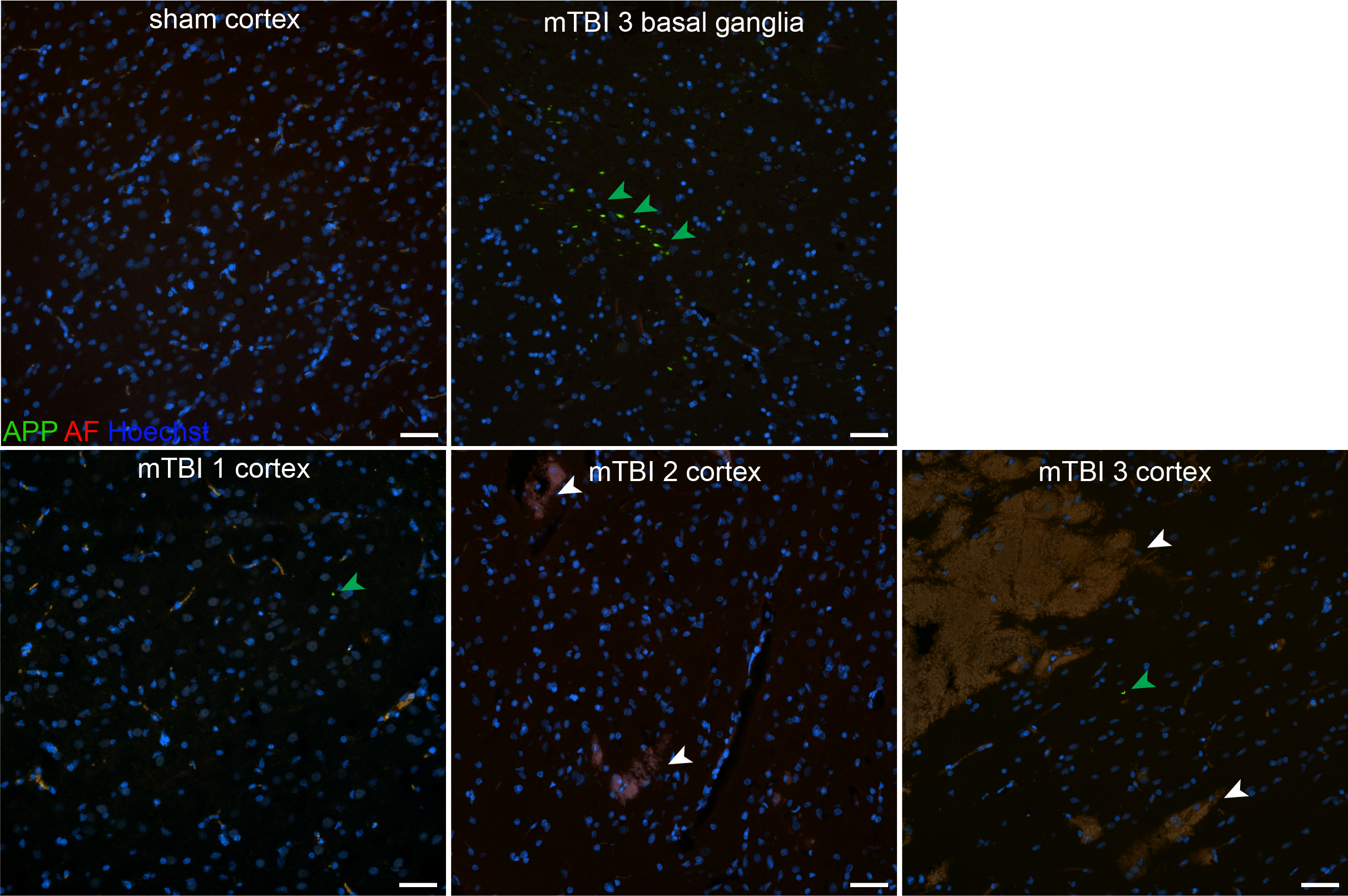

Supplement: Supplemental data [file Suppl_FigureS3.tif]
